# S11-3: Enhancing the reach of PA promotion among socially disadvantaged groups: lessons learned

**DOI:** 10.1093/eurpub/ckae114.248

**Published:** 2024-09-26

**Authors:** Kirsten Verkooijen, Daniëlla van Uden, Güven Alarslan, Dico de Jager, Annemarie Wagemakers

**Affiliations:** Wageningen University and Research, Wageningen, the Netherlands; Wageningen University and Research, Wageningen, the Netherlands; Wageningen University and Research, Wageningen, the Netherlands; Wageningen University and Research, Wageningen, the Netherlands; Wageningen University and Research, Wageningen, the Netherlands

## Abstract

**Purpose:**

People in socially vulnerable positions constitute a diverse group that face a variety of life challenges, such as low income, mental illness, homelessness, and social isolation. Participation in physical activity (PA) programs could help these people by strengthening resources, such as social support and mental health, that help them cope with these challenges. Several PA programs have been developed specifically for this purpose. However, the reach of these programs is often suboptimal. The aim of this research is to find (potentially) effective strategies to enhance the reach of PA promotion among socially disadvantaged groups.

**Methods:**

Qualitative data from three, past and ongoing, research projects on PA programs for socially disadvantaged groups were combined and analysed to extract important lessons with regards to effective strategies that enhance program reach. The data were gathered as part of the process and effect evaluation of these projects and involved interviews with program participants, program providers, and other relevant stakeholders.

**Results:**

Several effective strategies were identified to enhance the reach of PA programs among socially disadvantaged groups. At the organisational level, intersectoral collaboration was found to be very important for the recruitment of participants. At the program level, family-oriented programs showed potential to increase program reach as well as working with experts-by-experience and including the target group in the planning and design of activities. With regard to the latter strategy, providing meaningful involvement at the individual level appeared key.

**Conclusions:**

Notwithstanding the possible high quality of PA programs designed specifically for socially disadvantaged groups, their success is highly dependent on their reach among targeted participants. Further research into the effective strategies that increase program reach is warranted to inform and support practitioners involved in the design, implementation or organisation of these programs.

**Support/Funding Source:**

The projects were funded by ZonMW and NWO (Dutch organisations for scientific research).

